# Pronoun Interpretation in the Second Language: Effects of Computational Complexity

**DOI:** 10.3389/fpsyg.2017.01236

**Published:** 2017-07-21

**Authors:** Roumyana Slabakova, Lydia White, Natália Brambatti Guzzo

**Affiliations:** ^1^Modern Languages and Linguistics, University of Southampton Southampton, United Kingdom; ^2^Linguistics, University of Iowa, Iowa City IA, United States; ^3^Linguistics, McGill University, Montreal QC, Canada

**Keywords:** pronoun interpretation, referential antecedents, quantified antecedents, reduced pronouns, computational complexity, Binding Principle B

## Abstract

Children acquiring their native language (L1) have been reported to have greater difficulty in interpreting pronouns than reflexives. In addition, they are less accurate when pronouns refer to referential antecedents than to quantified antecedents, and when they hear full pronouns as opposed to reduced pronouns. We hypothesize that similar difficulties of interpretation will occur for (non-advanced) second language (L2) learners, due to an elevated computational burden, as argued for L1 acquisition by Reinhart (2006, 2011). We report on an experiment with adult learners of English (L1s French and Spanish), using a truth-value judgment task. Participants interpreted reduced and full pronouns bound by referential and quantified antecedents in aurally presented test sentences. The learners’ performance is affected by type of pronoun and antecedent. When a referential antecedent is combined with a full pronoun, learners’ accuracy is significantly lower. These results are in line with Reinhart’s analysis of reference set computation in processing pronouns.

## Introduction

In research on second language acquisition, as in research on child language, there has been ongoing investigation of the nature of the linguistic competence achieved by learners, in the course of development as well as in the endstate. From early on, the claim has been that interlanguage grammars are systematic, conforming to the properties of natural language (e.g., Corder, 1967; Selinker, 1972; Adjémian, 1976; see also White, 2003). At the same time, it is clear that other factors may impinge, such that second language learners/speakers (henceforth L2ers) show non-native performance even when their competence can be demonstrated to be native-like. For example, there have been proposals that L2ers are not able to access full representations when parsing (the Shallow Structure Hypothesis) (Clahsen and Felser, 2006); there have been proposals that L2ers may have difficulties integrating syntactic knowledge with discourse requirements (the Interface Hypothesis) (Sorace and Filiaci, 2006; Belletti et al., 2007); there have been proposals that morphological problems exhibited by L2ers reflect difficulties in accessing forms that are in fact present in the interlanguage lexicon, possibly under production pressure when speaking (the Missing Surface Inflection Hypothesis) (Haznedar and Schwartz, 1997; Prévost and White, 2000; see also Lardiere, 2000).

In this paper, we explore another possible factor which may affect L2 performance, namely computational complexity, identified by Grodzinsky and Reinhart (1993) and Reinhart (2006, 2011) as accounting for L1 acquirers’ relatively poor performance in interpreting referents for pronouns in certain contexts, compared to their performance on reflexives. We will show that L2ers have a problem with pronoun reference which is similar to (though not as severe as) child L1 acquirers; we suggest that the reason is the same, namely the computational complexity of the structure in question. This complexity may translate into an elevated processing load, though this is not directly tested in our study.

In order to explore this issue, we investigate the so-called Delay of Principle B Effect (DPBE) in adult learners of English. Principle B of the Binding Theory (Chomsky, 1981) constrains the distribution of pronouns (see below). Research on the DPBE in L1 acquisition has shown that children do not suffer from a representational deficit: Principle B is in fact present in child grammar but other factors sometimes cause children to fail to observe this principle. We suggest that the same holds true in L2 acquisition, at least in the case of learners who are not of advanced proficiency.

## Principle B And The Dpbe In Child Language

Pronouns (*him, her*, etc.) behave differently from anaphors like reflexives (*himself, herself*, etc.). In the typical case, the antecedent of an anaphor cannot occur in the same position as the antecedent of a pronoun.^1^ In particular, anaphors require their antecedents to be close (or *local*) whereas pronouns disallow this. Consider the English examples in (1) and (2):

(1) Mary_i_ thought [that Susan_j_ liked herself_j/^∗^i_]
(2) Mary_i_ says [that Susan_j_ likes her_i/k//^∗^j_]

In (1), the reflexive *herself* can only refer to the local antecedent, *Susan*, and not to the non-local antecedent, *Mary.* In (2), on the other hand, *Susan* is impossible as an antecedent for the pronoun, whereas *Mary* (or anyone else of female gender mentioned in the previous discourse) is a possible antecedent.

To express these relationships, Chomsky (1981) formulated Principles A and B of the Binding Theory, presented, in simplified form, below, where *local* means roughly “in the same clause”:

(3) Principle A: a reflexive must take a local antecedent.
(4) Principle B: a pronoun may not take a local antecedent.

In other words, Principle B renders local antecedents ‘inaccessible’ to pronouns.

It turns out that acquisition of pronouns, particularly with respect to choice of antecedents, presents rather distinctive challenges for children acquiring their first language (L1). In the acquisition of English and many other languages, a well-known and robust phenomenon known as the DPBE has been reported (Jakubowicz, 1984; Crain and McKee, 1985; Chien and Wexler, 1990; Koster, 1993; Avrutin and Thornton, 1994; Thornton and Wexler, 1999; among many others). In a nutshell, children are often at chance when interpreting sentences with pronouns, at stages when they have no problem in interpreting reflexives. In particular, they sometimes mistakenly assume that pronouns, like reflexives, can take local antecedents.

Delays in acquiring accuracy on pronouns have been observed cross-linguistically, for Dutch (Philip and Coopmans, 1996), Hebrew (Friedmann et al., 2010), Icelandic (Sigurjónsdóttir, 1992) and Russian (Avrutin and Wexler, 1992) but not for languages which have clitic pronouns (Spanish: Baauw et al., 1997; Baauw, 2002; Baauw and Cuetos, 2003; French: Zesiger et al., 2010; Italian: McKee, 1992; Greek: Varlokosta, 2002).

There is a further relevant finding in the literature, relating to whether the antecedent is referential (referring to a particular individual, e.g., Mama Bear) or quantificational (referring to some quantified group, e.g., *every bear*). Chien and Wexler (1990) found that 6-year-old children were much more accurate with quantified antecedents than with referential, mostly rejecting local antecedents for pronouns in the former case (84% rejection) while rejecting them in the latter only around 50% of the time. This finding has come to be known as “the quantificational asymmetry” in the interpretation of pronouns.

More recently, an additional asymmetry has been reported. Hartman et al. (2012) compared performance on fully pronounced versus phonologically reduced pronouns with referential antecedents, such as (5).

(5) I think… Cow washed ’m.

Hartman et al. (2012) used a truth-value judgment task (TVJT), in which participants saw stories acted out with toys, each story being paired once with a full pronoun test sentence and once with a reduced pronoun. In their experiment, children’s correct rejections of local antecedents for full pronouns were around 53% (similar to findings by Chien and Wexler, amongst others); on the other hand, rejection of local antecedents for reduced pronouns was significantly higher, at 80.6%.

To summarize so far, child language research has established that there is a DPBE in children’s comprehension. However, children are less accurate with pronouns referring to referential antecedents than with pronouns where the antecedent is quantified. Furthermore, a full pronoun versus reduced/clitic pronoun asymmetry is attested. Accuracy with quantified antecedents and with reduced pronouns suggests that Principle B is indeed operative and that some other explanation is required to account for the problematic cases.

### Toward an Explanation: Accidental Coreference

We turn now to an explanation of why pronoun reference should be particularly difficult to acquire, proposed by Grodzinsky and Reinhart (1993) and Reinhart (2006, 2011). There are two ways in which a pronoun and its antecedent can be associated. In addition to variable binding of pronouns (as regulated by Principle B), accidental coreference is also possible (Sag, 1976; Evans, 1980; Grodzinsky and Reinhart, 1993; Heim, 1993; Williams, 1977). In very specific contexts, a pronoun can in fact take a local antecedent. Such cases are heavily dependent on repetition and special intonation.

(6) A: Is that speaker Zelda?
  B: She must be. She praises her to the sky.
(7) From the movie “Side effects” (2013), directed by Steven Soderbergh, spoken by a psychiatrist to explain an accident with a patient:
“The patient blamed me. The patient’s wife blamed me. The patient’s children blamed me. Even I blamed me.”

These examples ostensibly violate Principle B, since the pronoun and its antecedent are in the same clause. Linguists have dealt with this problem by assuming that different indices have in fact been assigned to the pronoun and the antecedent; they just happen to refer to the same person, as shown in (6B’):

(6B’) She_i_ must be. She_i_ praises her_j_ to the sky. where i = j accidentally.

The assumption, then, is that, in interpreting pronouns, two derivations have to be constructed and compared. Reinhart (2006, 2011) calls this phenomenon “reference set computation” and invokes it as an explanation of other linguistic phenomena, such as Focus and scalar implicatures. Pronoun interpretation is computationally more complex than anaphor computation, for which only one interpretive mechanism exists, namely variable binding. As far as child language is concerned, Grodzinsky and Reinhart (1993) and Reinhart (2006, 2011) argue that the necessity for reference set computation with pronouns taxes children’s working memory resources; more specifically, reference set computation “relies heavily on the ability to store and perform further computation on temporary outcomes” (Reinhart, 2011: 168). On this account, when trying to interpret pronouns, children sometimes give up and pick an interpretation at random. This difference in computational complexity accounts for children’s roughly 50% accuracy on pronouns and their superior accuracy on reflexives.

This account also explains children’s accuracy with quantified antecedents, since these are subject only to variable binding, no accidental coreference being possible in such cases. The account has also been used to explain children’s accurate performance on pronouns in languages with clitics. According to Avrutin and Wexler (1992), accidental coreference is unavailable with clitic pronouns, because clitics are referentially deficient, in the sense that they are always bound variables (see also Baauw and Cuetos, 2003). This is suggested by the fact that clitics cannot be used in isolation, cannot receive focal stress, and cannot be used deictically with a pointing gesture. Children learning languages with clitics do not consider an accidental coreference derivation because of the requirement that the clitic is always coindexed with its antecedent, and so they are more accurate than children learning languages with strong pronouns, which are free to take on accidental coreference. English phonologically reduced pronouns, such as ’*m* for *him*, are similar to clitics in this respect.

To summarize so far, children’s greater success with quantified antecedents and reduced pronouns can be explained if children engage in reference set computation (deciding between binding and accidental coreference) only with full pronouns and with referential antecedents. In other words, computational complexity rather than lack of linguistic knowledge is the source of their difficulties. The account is potentially extendable to adult L2 learners.

## Pronoun Interpretation In L2 Acquisition

The issue of potential computational complexity, as defined by Reinhart and colleagues with respect to pronouns, has not been addressed in L2 acquisition. While there are a number of studies on the L2 acquisition of reflexives and their antecedents, less is known about pronouns. If the difference in accuracy in determining antecedents for pronouns and reflexives is computationally based, it is logical to assume that the same dissociation between pronouns and anaphors may arise in L2 acquisition as well. However, additional factors come into play in adult L2 acquisition. First, adult learners, having a fully developed computational system for their L1, may not display a big contrast between pronoun and anaphor interpretation accuracy in the L2, because they have learned to compute these meanings as children in their L1. Second, all languages have personal pronouns, in some cases taking the form of clitics, so L1 transfer into the L2 is possible, including transfer of requirements on possible antecedents. These two factors could aid learners in acquiring pronoun reference, and may obscure any computational effects that arise in the course of acquisition. Indeed, in the past, the understanding was that there are no significant problems with pronoun interpretation in L2 acquisition as far as Principle B is concerned (White, 1998).

Nevertheless, a new look at this phenomenon is warranted. First, the predictions made by the computational complexity account extend naturally to lower proficiency L2 learners, who may exhibit greater signs of struggling with pronoun interpretation than more experienced learners. Furthermore, new research using psycholinguistic techniques such as eye tracking (Kim et al., 2015) has already suggested that the processing of pronouns differs from the processing of anaphors, at least for Korean speakers of L2 English.

We turn now to a summary of previous research on pronoun interpretation in L2 as it relates to Binding Principles A and B. There has been extensive research on Principle A, looking at properties of reflexive pronouns, and focusing in particular on cross-linguistic differences that might come into play when the L1 and L2 differ with respect to whether long-distance antecedents are permitted (e.g., Finer and Broselow, 1986; Hirakawa, 1990; Thomas, 1995). There has been less work on Principle B. A few studies are relevant, either implicitly or explicitly, to the question of whether or not there is a DPBE in L2 acquisition; in particular, there are studies that compare performance on Principles A and B, looking only at cases involving referential antecedents.

Finer and Broselow (1986) were among the first to look at acquisition of an L2 (English) which permits only local antecedents for reflexives by speakers whose L1 (Korean) permits long-distance antecedents. Results from their pilot study of Korean learners of English (*n* = 6) on reflexives are well known: in tensed clauses, only local antecedents for reflexives were accepted, whereas in non-finite clauses non-local antecedents were accepted 40% of the time. What is less well known is that this study also included an examination of pronouns with referential antecedents. Results show that this small group of L2 learners accepted local antecedents for pronouns 46% of the time in tensed clauses and 21% of the time in non-finite clauses.^2^ In other words, if we consider only tensed clauses, they were much more accurate on interpretation of reflexives than pronouns, suggesting (indirectly) a possible DPBE.

Lee and Schachter (1997) argue for windows of opportunity in L2 acquisition, proposing that there are sensitive periods for L1 and L2 acquisition, such that certain properties cannot be successfully acquired before the onset of the sensitive period or after the end of it. Lee and Schachter tested this claim by looking at the L2 acquisition of Binding Principles A and B by Korean-speaking learners of English, with different ages of onset for the acquisition of English. Participants were tested on properties of reflexives and pronouns by means of a TVJT. Learners fell into various age categories at time of testing. The youngest groups (6–7 and 8–10 year olds) performed better on reflexives than on pronouns, consistent with the idea that the windows of opportunity open at different times for these two principles, and also consistent with a DPBE.

White (1998) investigated pronoun interpretation by Japanese-speaking and French-speaking learners of English, of high intermediate proficiency, hypothesizing that adult learners would not show problems with pronouns, on the assumption that difficulties with pragmatics, processing or computation, argued to account for the difficulties of children, would not arise for adults. Results from a TVJT show that the L2 groups appropriately rejected local antecedents for pronouns. In other words, there was no evidence of a DPBE in the groups as a whole. However, there were three participants (out of 28), one francophone and two Japanese speakers, who consistently accepted local antecedents for pronouns.

Two recent studies investigated anaphor and pronoun interpretation in L2 acquisition using eye tracking. Patterson et al. (2014) tested advanced German-speaking learners of English, to determine whether they know that a local antecedent for a pronoun is ‘inaccessible’ according to Principle B. In their experiment 2, participants read sentences which manipulated the gender of the potential antecedents. Native speakers and L2ers behaved alike: the non-local mismatch condition (sentences like *Jane remembered that John had taught him a new song*) resulted in longer reading times than the other conditions (*John remembered that Jane had taught him a new song; John remembered that Mark had taught him a new song).* While such results are consistent with the claim that L2ers are observing Principle B, the researchers question this interpretation. They added another experiment, involving clauses containing prepositional phrases (e.g., *Barry saw Gavin place a gun near him*). In such cases, the pronoun exceptionally allows a local antecedent (here *Gavin*), in violation of Principle B. Native speakers showed longer reading times when the object mismatched the pronoun in gender (e.g., *Barry saw Megan place a gun near him*), suggesting they were expecting a local antecedent for the pronoun. The L2ers, in contrast, showed longer reading times when the pronoun and the subject mismatched (e.g., *Megan saw Barry place a gun near him*). The researchers attribute the L2ers’ results not to Principle B but to “a general preference to link the pronoun to the matrix subject” (p. 15), and suggest that this also explains their success in experiment 2. We return to this issue in the discussion.

The second study to use eye-tracking, Kim et al. (2015), compared performance on Principles A and B. Assuming the Reflexivity Theory approach to binding (Reinhart and Reuland, 1993; Reuland, 2001, 2011), Kim et al. (2015) predicted that reflexives, being licensed syntactically, would be easier to interpret than pronouns, which in this framework require access to a pragmatic module in addition to syntax. The study used the visual world paradigm. Participants were adult native speakers of English as well as Korean-speaking learners of English, of intermediate to advanced proficiency.

Participants had to manipulate various cartoon characters displayed on the screen, in accordance with auditory instructions. With a mouse click, a character could be picked up and moved along a trajectory to a goal. Results were calculated in terms of the correct movement of the characters toward a potential antecedent as well as by the speed of eye fixation onto the place where the character had to be moved. Results indicate that when they heard a sentence with a pronoun such as *Look at Goofy. Have Mickey touch him*, the native speakers overwhelmingly chose the antecedent to be Goofy. The learners also predominantly chose Goofy as the antecedent; however, they also incorrectly chose Mickey as a possible antecedent 24% of the time, suggesting a DPBE effect, since they were totally accurate in the case of reflexives.

Furthermore, comparing the time it took the participants to start looking at the subject of the test sentence when they heard the lead-in sentences, the native speakers looked at the subject character (Mickey) no more in the pronoun condition than in the name condition (*Have Mickey touch Donald*). The L2 learners’, however, looked at Mickey significantly more in the pronoun condition, suggesting that they were considering Mickey as a potential antecedent. There was also a proficiency effect, in the sense that the lower proficiency learners took much more time to resolve the antecedent issue. The researchers concluded that the learners interpreted reflexives in a nativelike way, but demonstrated much more inaccuracy, hesitation and time delays when processing pronouns.

Few L2 studies have compared performance on referential and quantificational antecedents. One exception is Marinis and Chondrogianni (2011) who investigated the comprehension of reflexives and pronouns by children who are sequential bilinguals (L1 Turkish, L2 English). These children (mean age 7.8, ranging from 6.2 to 9.9) were compared to L1 acquirers of English (mean age 7.5, ranging from 6.0 to 9.0). The task, once again, was a TVJT. Test items included reflexives and pronouns; antecedents were referential or quantificational. While Marinis and Chondrogianni do not directly compare performance on reflexives with performance on pronouns, they do show that the bilingual children performed like the monolinguals on reflexives and were less accurate than monolinguals on pronouns, which suggests that Principle B was more problematic for them than Principle A. Both groups showed a quantificational asymmetry in the case of pronouns.

Before turning to our own study, we briefly mention a different kind of approach, namely the Interface Hypothesis (Sorace and Filiaci, 2006), which also predicts problems with pronoun interpretation in L2. Sorace and Filiaci (2006) and Belletti et al. (2007) report that advanced and near-native speakers of L2 Italian occasionally overuse overt subject pronouns in contexts where null pronouns would be preferred by native speakers. They attribute this overuse to problems at the syntax-discourse interface, namely a failure to fully appreciate the discourse requirements on overt pronouns, which imply a change in topic, unlike null pronouns which indicate topic continuity. The work of these researchers has focused on interpretation of subject pronouns, where Principle B is not at issue. Nevertheless, there are some commonalities in that processing problems have been suggested as an explanation (Sorace, 2011, 2016), a point we return to in the discussion.

The research described above suggests that all might not be well when it comes to pronoun interpretation in the second language. In the following section, we report on an experiment to investigate whether or not there is a DPBE effect in L2 and, if so, whether it is attributable to computational complexity. Our experiment does not focus on the comparison between anaphors and pronouns but instead on the interpretation of reduced versus full pronouns, and on the quantificational asymmetry with full pronoun antecedents. To anticipate the findings, we will show that learners of L2 English experience difficulties with pronoun interpretation. However, this only happens when full pronouns are combined with referential antecedents. In addition, learners’ interpretations are constrained by their level of proficiency in English. These findings are consistent with the assumption that computational complexity of the kind envisaged by Reinhart and colleagues is implicated.

## The Present Study

### Predictions

In section “Principle B and the DPBE in Child Language,” we presented the well-known delay in the correct interpretation of pronouns by children. As already discussed, we follow Grodzinsky and Reinhart (1993) and Reinhart (2006, 2011) in assuming that the DPBE reflects difficulties due to computational complexity caused by having to determine whether or not accidental coreference comes into play. We expect a similar difficulty of interpretation for L2ers, at least at lower levels of proficiency, attributable to the need to compute accidental coreference in the L2. Since accidental coreference is not possible with reduced pronouns or with quantified antecedents, we predict that learners will have difficulties only in cases where a full pronoun takes a referential antecedent. To investigate this prediction, we set out to establish whether learners of English with French or Spanish as their native languages correctly interpret sentences with reduced and full pronouns bound by referential and quantificational antecedents.

As discussed above, English is a language which has both strong and weak (phonologically reduced) forms of object pronouns (such as *him* versus *’m*). In contrast, French and Spanish are languages with object clitic pronouns, which differ in a number of respects from strong pronouns (see Kayne, 1975, for French). For example, as mentioned above, clitics cannot occur in isolation and are unstressed. They also differ from strong pronouns in their syntactic positions: object clitics are preverbal when the verb is finite. Spanish and French differ somewhat with respect to placement of clitics with non-finite verbs. We put these differences to one side as our test items only include finite verbs and the position of object pronouns is not under investigation. Given the similarities between French and Spanish with respect to object clitics, we do not expect differences in response patterns based on L1.

### Participants

A hundred and twenty-five individuals participated in two experiments: 65 in the Full Pronoun experiment and 60 in the Reduced Pronoun experiment. They comprised two groups of English native speakers, mostly recruited in Montreal, QC, Canada, and Southampton, United Kingdom, and four groups of learners of English with French or Spanish as their native languages, recruited and tested in Montreal. See **Table 1** for details.

**Table 1:** Participants in the two experiments.

	Full pronoun experiment			Reduced pronoun experiment		
	*n*	Female	Mean age	*n*	Female	Mean age
Native speakers	20	11	26.7	19	9	28.9
French-speaking L2ers	28	18	27.7	22	15	34.5
Spanish-speaking L2ers	17	10	28.7	19	10	32.5

The learners in both experiments had similar profiles. Most of them reported that they started learning English in a school setting (82.6%). The average age at which learners started to acquire English was 11.2, most of them between the ages 10 and 18 (60.5%). The majority of the learners were living in Montreal, QC, Canada, for work or study purposes. Some indicated having some knowledge of other languages (including French in the case of the native speakers of Spanish). Seven learners reported that they were taking English classes at the time of their participation in the experiment.

Testing took place individually (or in small groups in the case of native speakers) in a quiet lab. Participants took about half an hour to do the test (plus about 10 min for the proficiency test, in the case of the learners) and were remunerated for their participation.^3^

### Proficiency Test

Learners’ proficiency in English was assessed through an adapted version of the Oxford Test of Proficiency. The test included 40 grammar-based multiple-choice items, with a maximum score of 40. Learners’ mean proficiency scores for the two experiments are similar: 29.1 for the reduced pronoun experiment (range: 17–39), and 29.2 for the full pronoun experiment (range: 13–39). As will be discussed in the next section, we treat proficiency as a continuous variable.

### Truth Value Judgment Task (TVJT)

The TVJT (Crain and McKee, 1985; Gordon, 1996; Crain and Thornton, 1998) tests a speaker’s ability to evaluate interpretations of test sentences in controlled contexts/scenarios. The participant must decide whether a test statement is True or False as a description of a particular situation. A fundamental requirement of such tasks (Crain and Thornton, 1998) is that the story renders a grammatical reading false; consequently, only responses to stimuli expecting the answer False are considered to be truly informative of participants’ underlying grammatical competence. Furthermore, there is a Condition of Plausible Dissent (Crain and Thornton, 1998) or a Disputability Requirement (Conroy et al., 2009). The Condition of Plausible Dissent is satisfied if the grammatically inaccessible antecedent has been under consideration and is a genuine potential outcome of the story that almost comes to pass but in the end does not. This requirement ensures that the decision in the TVJT is taken on the basis of grammar, rather than the pragmatics of the story.

There is a further requirement, specific to TVJTs probing pronoun interpretation (Elbourne, 2005; Conroy et al., 2009): the Availability Requirement. Elbourne (2005) critiqued previous experiments for not making the antecedent sufficiently prominent in the story’s discourse. Only if children reject an available and prominent antecedent can we be certain that it is the child’s grammar, and not the discourse context, that is responsible for the attested interpretation. Following Conroy et al. (2009), we make sure this requirement is obeyed by including stories which mention groups of characters that are performing both reflexive and transitive actions. Our TVJT conforms to Conroy et al.’s (2009) recommendation that all characters mentioned in the story are sufficiently individuated to be considered as possible referents. In addition, all stories mentioned multiple characters so that the stories in the quantified antecedent condition did not involve more characters than stories in the referential antecedent condition. In the test conditions, each story is compatible with a reflexive as well as a pronominal interpretation.

In addition, we introduced another variable in our design. Within each condition (Referential antecedent, Quantified antecedent, filler), 4 sentences expected a True answer and 4 a False answer. Only the False-answer test sentences obey the above-mentioned TVJT design requirements; those expecting the True-answer serve as additional fillers.

We did not vary the factor quantified versus referential antecedent within items, because it was difficult to construct plausible stories that would fit both types of antecedents. We also did not vary the factor reduced versus full pronouns within participants, because we were concerned that a response bias or confusion might have been introduced if learners were exposed to both types of pronouns.

In what follows, we examine some representative context stories and explain how they satisfy or fail to satisfy the Requirements of Disputability and Availability. It is important to keep in mind that the contexts were presented visually in writing (on a computer screen) and aurally; test sentences were presented only aurally, since it was crucial that participants heard the form of the pronoun (full or reduced), rather than reading it.^4^ Each story was followed by a test sentence with either a reduced pronoun or a full pronoun, depending on the experiment.

A referential condition story with an expected False answer is exemplified in (8).

(8) Example from the referential condition with the expected answer ‘False’.
  Tom, Helen, and Harry were going to a soccer party. Prizes were going to be given out for the best spray-painted logo. They all sprayed the logo of their favorite soccer teams on their arms. Tom badly wanted to win the competition, so he asked his friends to help him make his logo even better. Helen refused to help because she wanted to win as well. Harry wanted to help Tom, but he had no spray-paint left.
    *Harry sprayed ’m*. (Reduced pronoun experiment) T F
    *Harry sprayed him*. (Full pronoun experiment) T F

The anaphoric (local, co-referential) reading (Harry sprayed himself) is available in this story, because all the three characters sprayed the logo of their favorite teams on themselves. The non-coreferential (non-anaphoric) interpretation (Harry sprayed Tom) is potentially available and under consideration, but in the end does not come to pass because there is no paint left. Thus the requirement of Disputability is satisfied.

In order to consider the requirement of Availability further, we compare this referential condition story with a quantificational condition story such as the one in (9), in which the expected answer is also False.

(9) Example from the quantificational condition with the expected answer ‘False’.
  Jim, Jack, and Bert always drive to college, each of them using his own car. Their friend John doesn’t own a car so Jim, Jack, and Bert all agreed to drive him to school. But this week, on Monday Jim forgot to pick John up. On Tuesday, Jack overslept and drove to class alone. Only Bert was true to his word and drove John to school on Wednesday.
    *This week, every student drove ’m to school* (Reduced pronoun experiment) T F
    *This week, every student drove him to school.* (Full pronoun experiment) T F

In parallel with the test item in (8), the anaphoric interpretation in (9) is available and prominent, because the three characters, Jim, Jack and Bert, always drive to school, each one using their own car, hence they drive themselves. The non-anaphoric interpretation (*Every student drove John to school*) is potentially under consideration and actually promised, but it never comes to pass due to highly individuated circumstances. Finally, the available propositions evaluated by the participants are closely matched in the stories in (8) and (9).

Let us now consider a True-answer story from the referential condition as in (10).

(10) Example from the referential condition with the expected answer ‘True’.
  Christopher, Mary, and Ben work in a bakery. Christopher and Mary bake bread and pastries and Ben sells them. Mary always wears an apron but Christopher does not. At the end of each day, Christopher is very dusty from all the flour. Ben dusts his friend’s clothes and hair off until Christopher is completely clean.
    *Ben dusts ’m off*. (Reduced pronoun experiment) T F
    *Ben dusts him off*. (Full pronoun experiment) T F

In this story, the anaphoric interpretation is missing: Ben never dusts himself off. The requirement of Disputability is also not obeyed: there is nothing to dispute since the action is actually confirmed. In addition to violating the TVJT requirements, these stories are easier to interpret, since the correct pronominal interpretation (the non-anaphoric one) is rather prominent. In addition to stories with referential or quantificational antecedents, the experiment included stories followed by test sentences containing full NPs in object position. These items were also treated as fillers; see (11).

(11) Example of filler story with a full NP in object position in the test sentence.
  Anne, Margo, Celia and Rita find an old empty house and spend all day playing inside. They get covered in dust. They try to clean the dust off themselves but Anne is no good at it. Anne asks Rita to help her, but Rita is too tired. Celia has already gone home. In the end, Margo agrees to help and does a great job.
    *Margo cleans Anne.* T F

To summarize, we have 8 test items in each experiment (responses where the expected answer is False), and 16 filler.^5^ In other words, each experiment (reduced or full pronouns) comprised 24 story–test sentence combinations: 8 test items expecting False answers, 8 fillers expecting True answers and 8 fillers with full NPs in object position, with answers that were true or false. Within the items involving pronouns, 4 had referential and 4 had quantificational antecedents. The context stories were identical in the two experiments. Test items differed, involving the full pronoun *him* in one experiment and the reduced pronoun *’m* in the other.^6^ Each participant was tested on all 24 story-sentence combinations within one experiment; no participant undertook both experiments. The presentation software (SurveyGizmo) randomized the order of item presentation for each participant.

### Statistical Analysis

We modeled learners’ responses for the target test items using a multilevel logistic regression with random effects (glmer() in R; R Development Core Team, 2017). The maximal converging model included the following predictors: *native language* (French or Spanish), *proficiency score* (continuous variable), *antecedent* (referential or quantified), *pronoun* (full or reduced), and the interaction between *antecedent* and *pronoun*.^7^ We included this interaction given the hypothesis that any inaccuracy will be the result of computational complexity and is, therefore, dependent on both type of antecedent and type of pronoun. In addition, the model included a by-item random intercept and a by-speaker random slope for antecedent, to account for the variation among test items and the variation among speakers with regard to antecedent, respectively.

A separate logistic regression with the same predictors (both main effects and random effects) was run to verify whether learners’ responses to the True answer fillers were affected by any of the predictors included in the analysis. In order to compare the accuracy of learners and native controls, we performed two chi-square tests, one comparing the groups with respect to their accuracy on the fillers, the other comparing their accuracy on the target items.

### Results

Participants either took part in the experiment that included reduced pronouns or the experiment that included full pronouns. As described above, the target items were those for which False answers were expected, with two types of fillers: items for which True answers were expected and items containing full NPs instead of pronouns in object position. Participants’ accuracy on both types of fillers was high (**Table 2**); controls were more accurate than learners on fillers (χ^2^ = 23.1, *p* < 0.0001). The logistic regression for the True answer fillers indicates that learners’ accuracy is not conditioned by antecedent, pronoun, or the interaction between antecedent and pronoun (*p* > 0.05).

**Table 2:** Mean accuracy (%) on fillers by group and experiment (reduced or full pronouns).

	Controls		Learners	
	Reduced pronouns	Full pronouns	Reduced pronouns	Full pronouns
Full NPs	98.6%	98.6%	93.2%	94.6%
True answers	92.05%	95.75%	82.85%	87.2%

We now turn to participants’ accuracy on the target items. **Table 3** shows mean accuracy scores on items expecting False as the answer, by pronoun and antecedent.

**Table 3:** Mean accuracy scores (in %) by group, pronoun, and antecedent.

	Controls		Learners	
	Reduced pronouns	Full pronouns	Reduced pronouns	Full pronouns
Quantified antecedents	100%	95.8%	87.1%	90.5%
Referential antecedents	98.6%	97.2%	91.4%	82.7%

**Table 3** shows (a) that the controls perform at ceiling while the L2ers are in general less accurate than controls (χ^2^ = 24.2, *p* < 0.001); (b) in the case of full pronouns, the L2ers are more accurate with quantified antecedents than with referential antecedents; and (c) the L2ers are the least accurate with full pronouns taking referential antecedents. Thus, while the controls’ performance is not affected by type of pronoun or antecedent, the L2ers’ performance is.

**Table 4** shows the estimates of our statistical model for L2ers’ performance on the target items. A positive estimate (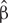) indicates that the predictor in question is associated with an increase in accuracy.

**Table 4:** Coefficient values, standard error (SE), *z-*value (Wald test), and *p-*value for predictors in the statistical model.

Predictors	Estimate ( 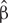 )	SE	*z*-value	*p-*value
Intercept	3.36	0.65	5.1	<0.001
Native language (French)	-0.49	0.51	-0.96	0.33
Proficiency score	1.18	0.27	4.3	<0.001
Full pronoun	0.3	0.53	0.57	0.56
Referential antecedent	1.04	0.78	1.33	0.18
Referential antecedent ^∗^ Full pronoun	-1.76	0.64	-2.74	0.006

The results for *native language* indicate that, as expected, there is no significant difference between French-speaking and Spanish-speaking L2ers’ responses. On the other hand, learners’ performance improves significantly as their scores in the proficiency test increase. Each unit^8^ increase in proficiency test scores raises the odds of getting a right answer by a factor of 1.39 [exp(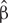)].

**FIGURE 1:**
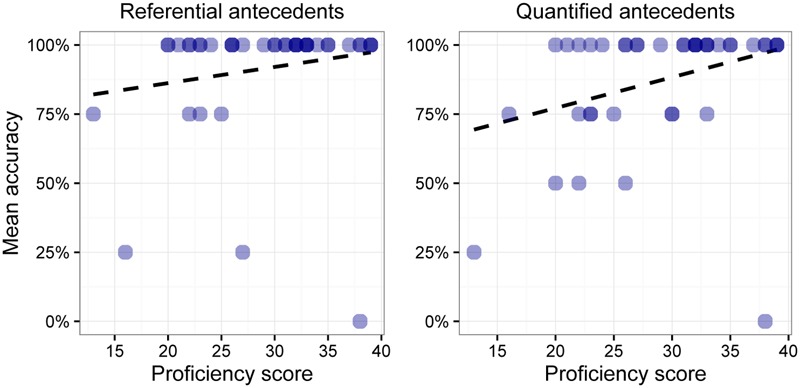
Individual accuracy with reduced pronouns.

**FIGURE 2:**
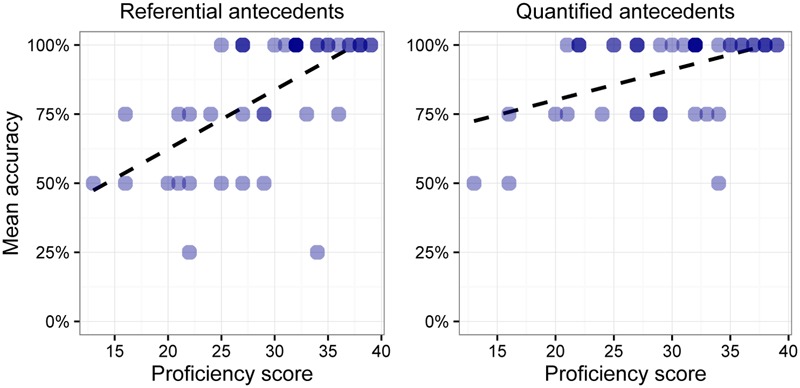
Individual accuracy with full pronouns.

**Figures 1, 2** show the L2ers’ mean accuracy on each of the four possible combinations of antecedent and pronoun. In each figure, the *x*-axis shows scores on the proficiency test while the *y*-axis shows learners’ mean accuracy in the task. The darker circles indicate a higher concentration of L2ers with a given mean accuracy and proficiency score. There are two patterns of note in these figures: (a) L2ers with a lower score on the proficiency test overall perform worse than learners with a higher score on the proficiency test, and (b) the combination of a referential antecedent and a full pronoun (left panel in **Figure 2**) yields a higher concentration of lower scores than the other possible combinations between antecedent and pronoun in the data, as indicated by the steeper slope of the trend line. In particular, problems do not arise with quantified antecedents or with reduced pronouns.

The model indicates that the interaction between *antecedent* and *pronoun* is significant: when a referential antecedent is combined with a full pronoun, learners’ accuracy goes down, as suggested by the trend lines in **Figure 2** and the values in **Table 3**. This interaction has a negative effect on L2ers’ accuracy, which is consistent with our hypothesis. *Pronoun* and *antecedent*, however, are not significant as main effects.

In summary, L2ers’ accuracy on test items is affected by *proficiency score* and the interaction between *antecedent* and *pronoun*: learners who are more proficient are overall more accurate, and learners’ performance is worse on the combination between referential antecedents and full pronouns. The next section discusses these results in light of our predictions.

## Discussion

Let us recap the predictions and significant findings of this study. We set out to evaluate pronoun reference by native speakers and L2ers, in the light of difficulties exhibited by L1 acquirers, the so-called DPBE. We evaluated L2ers’ interpretations in two experiments, identical except for the form of the pronoun: in one, participants heard full pronouns in the test sentences; in the other, they heard phonologically reduced pronouns. In both cases, test items involved quantificational and referential antecedents. Participants had to evaluate the truth of the test sentences in a TVJT with contexts presented in written and spoken form, and test sentences presented only aurally.

As far as we are aware, no study of L1 or L2 acquisition has looked at the combination that we investigated, namely a comparison of referential and quantified antecedents for full and reduced pronouns. This combination is essential to fully assess the potential role of reference set computation in learners’ determination of antecedents for pronouns. Given findings in the L1 literature that children have greater difficulties with pronouns with referential antecedents than with quantified antecedents, the so-called quantificational asymmetry, and greater difficulties with full pronouns than with reduced pronouns, we expected to find lower accuracy on referential antecedents but only in the case of full pronouns. This prediction was supported by the multilevel logistic regression results reported in the previous section.

As far as the lower proficiency L2ers are concerned, we observed greater accuracy on quantified antecedents than on referential antecedents with full pronouns, as can be seen in **Figure 2**. We also established greater accuracy with reduced pronouns versus full pronouns, in the case of referential antecedents; see **Table 3** and the left-hand panels of **Figures 1, 2**. Lower proficiency L2ers achieved relatively high accuracy in the reduced pronoun version of the experiment (**Figure 1**). The steeper slope on the left panel of **Figure 2** indicates that the learners are less accurate in the full pronoun-referential antecedent combination.

More advanced learners did not exhibit a quantificational asymmetry, nor did they manifest reduced accuracy with full pronouns, as can be verified by looking at the higher proficiency individuals in **Figures 1, 2**. They were able to identify the correct antecedents for all pronouns in each experiment. The same pattern was observed in the native speakers; see **Table 2**. These findings suggest that advanced and native speakers were essentially performing at ceiling.

Our findings are easily accounted for in terms of the computational complexity proposal of Grodzinsky and Reinhart (1993) and Reinhart (2006, 2011). These researchers argue that when the antecedent is a referential NP, children have to consider both variable binding as well as accidental coreference as possible routes to finding an appropriate referent. Constructing the reference set, keeping it in short-term memory and comparing the two derivations proves costly, and in the end children give up and choose an available antecedent at random. Quantificational antecedents, on the other hand, do not allow accidental coreference, and neither do reduced pronouns, hence the computational task becomes much simpler, and children are more accurate. The fact that our lower proficiency L2ers were least accurate on full pronouns with referential antecedents suggests that the same computational burden arises in L2 acquisition, although not perhaps to the same degree, since our participants performed above chance on these sentences, unlike children.

To reiterate, the child language discoveries of a quantificational asymmetry and a clitic advantage found parallels in the performance of non-advanced L2ers. The fact that these same participants are at ceiling with reduced pronouns suggests that they know how to interpret such pronouns. The fact that they are highly accurate with quantified antecedents suggests that full pronouns are not always problematic. In other words, our lower proficiency learners do not have an underlying problem with all pronoun interpretation, but only with the difficult-to-compute cases, in consort with 6-year-old children acquiring English. No other theoretical account can explain the child—L2 learner parallel behavior.

In this respect, it is instructive to review Patterson et al.’s (2014) findings, in order to see whether their analysis can explain our results. These researchers attributed the performance of the L2ers in their experiments to a general preference for the non-local matrix subject to serve as the antecedent for a pronoun, even when this was not in fact the case for native speakers (as in the exceptional sentence types). However, such an explanation cannot account for our results. Our participants sometimes chose a local referential subject as antecedent and did so differentially in the case of full versus reduced pronouns.

As discussed above, Sorace and colleagues (as described in Sorace, 2011, 2016) have also proposed that certain problems relating to L2 pronoun interpretation (instability and overuse of overt subject pronouns in languages like Italian) may be attributed to differences in available processing resources, rather than differences in knowledge representation. The suggestion is that bilingual processing is less efficient than monolingual processing, either because of difficulties in accessing and integrating different kinds of linguistic knowledge or because of the availability of fewer cognitive resources in general. In our account of computational complexity, we follow Grodzinsky and Reinhart (1993) and Reinhart (2011) in assuming that, as far as Principle B is concerned, the complexity relates to the fact that speakers have to compute and compare two linguistic derivations and ultimately reject one of them, which sometimes proves difficult or impossible for language learners. In other words, our definition of computational complexity is somewhat narrower than Sorace’s approach to availability or non-availability of certain processing resources. Nevertheless, we concur that an increased processing load is implicated in both cases; it is this processing load rather than representational difficulties that underlies the performance of our participants.

Coming back to our own findings, we must acknowledge two alternative explanations of the greater accuracy on reduced pronouns that we found. The first is that the L1s of our participants were French or Spanish, both languages with clitic pronouns, so participants could presumably have transferred the requisite knowledge that clitics do not allow accidental coreference from their native languages. In other words, their greater accuracy with reduced pronouns would reflect L1 transfer. On the other hand, if transfer is the main factor at work, it remains unexplained why participants had problems precisely in those areas where accidental coreference needs to be computed and rejected; given the L1s in this case, accidental coreference should not have been entertained at all and so no computational complexity should have arisen. In order to eliminate the possibility of transfer, a necessary next step will be to add participants whose L1 does not have clitic-like pronouns, in order to see whether they can recognize the clitic-like properties of English reduced pronouns, including the fact that the computational burden is decreased in such cases.

The second objection that might be raised to our study is that the English reduced pronoun *’m* can be ambiguous between *him* and *them*. Could it be that the participants interpret *’m* as *them*, then reject the sentence in stories like (8) for the wrong reason, accounting for their greater accuracy with reduced pronouns in the False scenarios? In fact, if this were the case, then one would expect inaccuracy (i.e., rejections) on the scenarios where the expected answer is True [see (10)], contrary to what was found. Clarification on this point could be provided by including an unambiguous reduced pronoun, such as *’r* (*her*) in subsequent studies. A related point is the possibility of participants not hearing the reduced pronoun at all, and treating the verbs as intransitive, e.g., *Harry sprayed* and *Every student drove to school.* In order to evaluate this possibility, we examined the eight verbs in our test. Only four of them could be used intransitively, suggesting that omission of the pronoun is not a likely explanation of our results. As pointed out above, the effects in our model take into account the possible by-item variation present in the data.

Another possible objection to our analysis here is that a computational burden would seem to imply a measurable processing cost but our experiment included only an untimed TVJT, a measure of interpretation, not processing. We concur with Sorace (2011: 20), who points out that it is a misconception to assume that processing cannot be addressed by means of offline tasks. The fact that lower proficiency participants in our study had a problem in interpreting ONLY those stimuli where a computational cost is implicated is already an indication of a processing cost. Furthermore, while children’s difficulties with pronouns have primarily been documented with comprehension studies, a number of studies have confirmed that the same contrast holds in online processing as well. For example, Clackson et al. (2011) conducted a visual-world eye tracking study on the processing of both reflexives and pronouns by 6-to-9-year-old English-speaking children. The results suggest that both adults and children experienced competition and interference when they had to consider two same-gender antecedents for pronouns, one grammatically permitted, namely the matrix subject, and one an inaccessible competitor antecedent, the embedded clause subject, in sentences such as *Peter was waiting outside the corner shop. He watched as Mr. Jones bought a huge box of popcorn for him over the counter*. However, adults were able to overcome this difficulty and provide accurate offline judgments, unlike children, whose judgments were significantly less accurate.

In SLA research, too, the recent eye tracking study of Kim et al. (2015) uncovered a sharp contrast in L2 learners’ treatment of reflexives and pronouns (see Pronoun Interpretation in L2 Acquisition), partially consistent with our offline findings [since Kim et al. (2015) used only referential antecedents and full pronouns]. Thus both interpretation and processing findings point in the same direction: pronouns are more difficult to process than reflexives, although individuals with higher processing resources are capable of accomplishing the necessary reference set computation.

Although we look at offline pronoun interpretation by L2 learners and establish lower accuracy for full pronouns with referential antecedents, our approach predicts processing difficulties even when the learners make the right choice (rejecting local antecedents for pronouns). Such behavior is already previewed in results from Clackson et al.’s (2011) adult native speakers, who demonstrated difficulties reflected in online measures but managed to compensate in offline measures. The higher computational burden is predicted to be reflected in longer reaction times, or greater hesitation, even when participants succeed in reference set computation. We leave this prediction for further research.

## Conclusion

We have looked at how a proposed computational burden has effects on linguistic performance, such that L2 learners occasionally and temporarily make inaccurate judgments as to referents for pronouns, parallel to the difficulty reported for L1 acquirers. That this is not an issue of inappropriate representation is demonstrated by L2ers’ accuracy with quantified antecedents and with reduced pronouns, in contrast to their performance on full pronouns with referential antecedents. Our findings take us beyond earlier L2 research on pronoun interpretation which has rarely looked at the quantificational asymmetry and never, as far as we are aware, at the differential status of the pronoun. Our results support the claim that a computational burden is implicated in L2 as in L1, and that this burden can be overcome—advanced L2ers do not differ from native speakers in their ability to select the appropriate antecedents for pronouns, even when they have to compute and reject accidental coreference.

In keeping with the research topic “Language acquisition in diverse linguistic, cognitive and social circumstances,” we have uncovered a similar pattern of behavior between children acquiring their native language and L2ers at lower levels of proficiency, despite considerable diversity in acquisition circumstances (age, cognitive capacities, input, etc.). The child–adult parallels with respect to difficulties in engaging in reference set computation and eventual success in this domain are noteworthy. At the same time, there are child–adult differences: adult L2ers do not experience as severe a difficulty as children (around 83% accuracy compared to 53%). This is not surprising, given that adults presumably have computational abilities that are superior to those of children. What is of interest is that being an adult is not sufficient to remove the computational burden altogether.

## Author Contributions

RS and LW contributed equally to the design of the experiment, the interpretation of the results, the discussion and the writing up of the article. NG performed the statistical analysis, wrote the results section and contributed to the interpretation discussion.

## Conflict of Interest Statement

The authors declare that the research was conducted in the absence of any commercial or financial relationships that could be construed as a potential conflict of interest.

## Footnotes

1 We exclude from consideration here anaphors which allow long-distance antecedents, as found in languages like Chinese, Japanese, or Korean, for example.

2 Finer and Broselow do not, in fact, discuss their results relating to pronouns, but they are available in an appendix.

3 Informed consent was obtained from all participants. The research program under which this project was conducted was reviewed by Research Ethics Board II of McGill University and is deemed to be in compliance with the ethical standards expected for research with human subjects (approvals: REB #451-0511 and #60-0715).

4 There were no pictures accompanying the text.

5 Hartman et al. (2012) have 4 test items expecting the answer False with full pronouns and 4 with reduced pronouns; they only tested referential antecedents.

6 Female characters were introduced in the stories but did not occur in the test items.

7 The underlined levels in parentheses are the reference levels for our predictors. Except for *native language*, where we do not expect to find any main effects, the reference levels for *antecedent* and *pronoun* were decided upon based on our predictions (see Predictions).

8 Given that the predictor *proficiency result* was scaled and centered, each unit here is equivalent to one standard deviation in the proficiency score (*SD* = 6.58).
